# Association Between Aflatoxin Exposure and Haemoglobin, Zinc, and Vitamin A, C, and E Levels/Status: A Systematic Review

**DOI:** 10.3390/nu17050855

**Published:** 2025-02-28

**Authors:** Naelijwa Mshanga, Sally Moore, Neema Kassim, Haikael D. Martin, Carolyn I. Auma, Yun Yun Gong

**Affiliations:** 1 School of Life Sciences and Bioengineering, The Nelson Mandela African Institution of Science and Technology, Arusha P.O. Box 447, Tanzania; neema.kassim@nm-aist.ac.tz (N.K.); haikael.martin@nm-aist.ac.tz (H.D.M.); 2 School of Food Science and Nutrition, Faculty of Environment, University of Leeds, Leeds LS2 9JT, UK; s.moore2@leeds.ac.uk (S.M.); c.i.auma@leeds.ac.uk (C.I.A.); y.gong@leeds.ac.uk (Y.Y.G.)

**Keywords:** aflatoxin, micronutrients, vitamins, minerals, humans

## Abstract

**Background:** Aflatoxin, produced by *Aspergillus flavus* and *Aspergillus parasiticus* fungi, contaminates a broad range of crops such as maize, nuts, and cotton. Aflatoxin exposure causes growth failure, immune suppression, and liver cancer. While several systematic reviews have assessed the link between aflatoxin exposure and growth development in humans, there is a lack of reviews on the associations between aflatoxin exposure and micronutrient levels/status. This review addresses that gap by compiling studies on the association between aflatoxin exposure and micronutrient levels/status in humans. **Methods:** A comprehensive search of the SCOPUS, PUBMED, EMBASE, and Web of Science databases was conducted, focusing on studies published between 2003 and 2023. Only English-language studies using urine, blood, serum, or plasma biomarkers were included to assess the exposure and outcomes. The risk of bias in these studies was evaluated using the Academy of Nutrition and Dietetics Quality Criteria for human studies. **Results:** Ten observational studies were included in the systematic review, which collectively reported the association between aflatoxin exposure and haemoglobin, zinc, and vitamin A, E, and C levels. This review suggests that aflatoxin exposure is associated with micronutrient deficiencies, such as anaemia (low haemoglobin levels (<11 g/dL)) in pregnant women and vitamin A deficiency in adults and children. **Conclusions:** This review highlights the link between aflatoxin exposure and micronutrient deficiencies, emphasizing the need for aflatoxin mitigation within micronutrient interventions. Future studies should focus on longitudinal and interventional research to establish causal relationships and assess the effectiveness of mitigation strategies. Additionally, further research is needed to explore the interaction between aflatoxin exposure and other potential confounding factors such as dietary patterns, socioeconomic status, and genetic predisposition.

## 1. Introduction

Micronutrients, such as iron, iodine, zinc, calcium, and vitamins C, A, E, B9, and B12, are essential nutrients, and their deficiencies can lead to severe and chronic diseases, significantly impacting human health and well-being [1]. The World Health Organization (WHO) has identified iron, vitamin A, zinc, and iodine deficiencies as micronutrients of public health concern, particularly in low- and middle-income countries [2]. The global prevalence of a deficiency in at least one of three micronutrients (iron, zinc, vitamin A) is estimated at 56% among preschool-aged children (equivalent to 372 million) and 69% among non-pregnant women of reproductive age (equivalent to 1.2 billion). Regionally, three-quarters of deficient preschool-aged children are concentrated in South Asia (99 million), sub-Saharan Africa (98 million), and East Asia and the Pacific (85 million). Among women of reproductive age, over half of those affected live in East Asia and the Pacific (384 million) and South Asia (307 million) [3].

Deficiencies in micronutrients, i.e., iron/anaemia, zinc, and vitamins A, C, and E, can lead to several health problems in women of reproductive age and children under five [4]. Vitamin A deficiency can led to night blindness and low immunity, hence increased susceptibility to infections [5], while vitamin E deficiency can increase oxidative stress in the body [6]. In addition, iron deficiency/anaemia can lead to fatigue, shortness of breath, impaired cognitive function, and poor growth [7], whereas zinc deficiency can cause delayed wound healing, skin diseases, and diarrhoea [8]. Micronutrient deficiencies can result from a poor intake of micronutrient-rich foods (e.g., fruits and vegetables), gastrointestinal diseases (e.g., diarrhoea, worm infections), which impair nutrient absorption and environmental factors (i.e., changes in climatic conditions can reduce the availability of micronutrient-rich foods) [9].

While many studies have identified various causes of micronutrient deficiencies, few have investigated aflatoxin as a contributing environmental factor. Aflatoxins, a toxin produced by the *Aspergillus flavus* and *Aspergillus parasiticus* fungi, contaminates foods such as maize, peanuts, oil seeds, and dried fruits during growth or storage [10]. A systematic review of the literature on the aflatoxin situation in Africa [11] has shown that all studies that assessed aflatoxin B1 (AFB1) in maize found a mean AFB1 of >5 μg/kg, which is above the European Union legal limit for AFB1. Additionally, studies using biomarkers to assess aflatoxin exposure in African populations found aflatoxin detectable levels in over 64% to 75% of individuals when breast milk, urine, and blood were assayed [12,13,14]. Aflatoxin exposure has been associated with a six-fold increase in stunting among under-five children, as observed in a cross-sectional study of 205 children in Kenya [15]. Furthermore, a meta-analysis of animal studies has reported a decrease in micronutrient levels in animals who were given a high amount of aflatoxin in their diet compared to animals with low or no aflatoxin in their diet [16].

Despite these findings, limited human studies have explored this association and those that did have relied on observational design. Therefore, this systematic review aimed to compile available evidence on the association between aflatoxin exposure on any micronutrient level/status using urine/blood/plasma/serum biomarkers, a connection that remains underexplored. By highlighting this association, the review underscores the importance of incorporating aflatoxin mitigation strategies into public health interventions aimed at improving micronutrient statuses.

## 2. Materials and Methods

Registry for Systematic Review: This systematic review was registered at PROSPERO (CRD42023458083).

To assess whether aflatoxin exposure is associated with any micronutrient level/status, a systematic review in human studies was undertaken following a predefined protocol published in the International Prospective Registration of Systematic Reviews (PROSPERO) with the identification number CRD42023458083. Moreover, the Preferred Reporting Items for Systematic Reviews with Meta-Analyses (PRISMA) [17] and Synthesis without Meta-analysis (SWiM) tools [18] were used as guidelines to conduct and report on this systematic review.

### 2.1. Search Strategy

Systematic literature searches were performed in March 2023 to identify peer-reviewed research published between January 2003 and March 2023 in human studies since a scoping search identified few studies assessing the relationship between aflatoxin B1 exposure and micronutrient levels/status. Electronic searches were conducted, and the search strategies for each database are detailed in Appendix A. Specifically, PubMed (Ovid) is shown in Table A1, EMBASE in Table A2, Scopus in Table A3, and Web of Science in Table A4. The search strategy was designed to fit the Population, Exposure, Comparison, and Outcome (PECO) framework as follows:

(a) Population: All human beings.
(b) Exposure: Aflatoxin exposure assessed by urine (Aflatoxin M1 (AFM_1_)), plasma, and serum biomarkers (Aflatoxin B1 albumin adducts (AF-alb)) in humans.
(c) Comparison: Low aflatoxin B1 exposure or without aflatoxin B1 exposure.
(d) Outcome: Any micronutrient level/status (i.e., normal or deficient) assessed by urine, serum, and plasma biomarkers.

### 2.2. Inclusion and Exclusion Criteria

Studies considered included those with either experimental or observational designs that examined the association between aflatoxin exposure and any micronutrient level/status in humans; published between 2003 and 2023; and that reported statistical measures of association (i.e., odds ratios, relative risk or correlation coefficients) regardless of geographical location, age, and population. This inclusive approach was coupled with the exclusion of studies that did not assess the association between aflatoxin exposure in relation to micronutrient status; those involving animals; those not written in English; those with incomplete or non-extractable data; and review and case reports.

### 2.3. Study Selection

Articles obtained from the database search were uploaded to Rayyan software V 1.5.0 for title and abstract screening. The first author (N.M.) screened the titles and abstracts against the inclusion and exclusion criteria, and where there were any disagreements, a discussion of whether to include an article or not was agreed upon by consensus between two reviewers (Y.Y.G and N.K). The full texts of the selected studies were retrieved and reviewed against the same inclusion and exclusion criteria. Discussions with other authors (N.K, S.M, C.I.A, H.D.M., and Y.Y.G.) resolved disagreements on inclusions and exclusions.

### 2.4. Data Extraction and Synthesis

Key data from articles passing the full-text review were extracted into an Excel template for this review. The extraction template captured information on study characteristics, analytical methods used to assess aflatoxin and outcome measurements. A narrative synthesis of the selected studies was formulated which included the author’s name, country, year of publication, study design, study population, sample size, analytical method used to assess aflatoxin exposure (i.e., Enzyme-Linked Immunosorbent Assay (ELISA), Radio Immunoassay (R.I.A.), and High-Performance Liquid Chromatography (HPLC)), and outcome measures (indicators related to micronutrients).

The standardized metrics used to assess the association between aflatoxin exposure and any micronutrient level/status were the odds ratio, correlation coefficient, and *p*-values. The statistical significance was assessed using three thresholds: *p* < 0.05, *p* < 0.01, and *p* < 0.001. A *p*-value less than 0.05 suggests that there is less than a 5% probability that the observed association occurred by chance, while *p* < 0.01 and *p* < 0.001 indicate even more substantial evidence against the null hypothesis. These varying levels of significance reflect the strength of the reported associations across studies.

Moreover, only studies with a low and moderate risk of bias and with 100 sample sizes and above were included in the main synthesis. The narrative synthesis was summarized in one table, whereas studies were organized based on the study population; thus, studies with children under ten years old (0–9 years), adults (>16 years), and pregnant women were grouped. Within each study population category, studies were arranged alphabetically.

### 2.5. Assessment of Aflatoxin B1 Exposure

The majority of the studies used the AF-alb biomarker to detect aflatoxin exposure, while only one study used the AFM_1_ biomarker in urine. The detection limits varied based on the analytical method used to detect AF-alb and AFM_1_. For the AFM_1_, the detection limit was >0.04 ng/mL in ELISA, while the detection limit for AF-alb was ≥0.01 pmol/mg, ≥0.4 pg/mg, and ≥3 pg/mg for R.I.A, HPLC, and ELISA analytical methods, respectively. These detection limits indicate the minimum amount of aflatoxin that can be reliably detected by each method, thereby influencing the sensitivity and accuracy of the results. The variation in each detection limit is due to the difference in the degree of specificity and sensitivity and the underlying principles that each analytical method carries [19,20,21].

### 2.6. Risk of Bias Assessment

The risk of bias (RoB) in human studies was assessed using the Academy of Nutrition and Dietetics Quality Criteria Checklist. This tool evaluates factors such as research question clarity, participant recruitment, group comparability, withdrawals, blinding, defined outcomes, statistical methods, and funding bias. Studies with mostly “yes” responses to key questions were rated as low RoB. If some critical answers were “no”, a moderate RoB rating was given. Studies with many “no” answers were rated as high RoB. The results were visualized using the Robvis software package V 0.3.0.

## 3. Results

**Figure 1:**
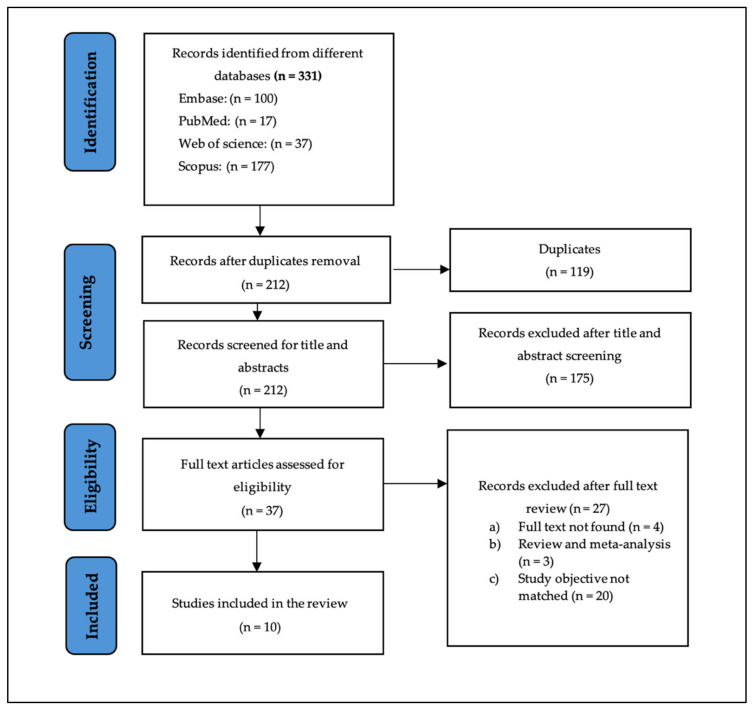
PRISMA diagram for the selected articles for systematic review.

Three hundred and thirty-one (331) studies were gathered from the four electronic databases, which were later reduced to 212 after removing duplicates (Figure 1). The remaining articles were screened for titles and abstracts, which resulted in removing 175 articles. The full texts of the 37 remaining articles were screened, and only 10 of those studies were included. The description of each study is presented in Table 1.

### 3.1. Study Characteristics

The number of studies that explored the association between aflatoxin exposure and each specific micronutrient was as follows: vitamin A (n = 6), vitamin E (n = 4), vitamin C (n = 1), Zinc (n = 3), and anaemia (assessed through haemoglobin levels) (n = 4).

### 3.2. Quality Appraisal

**Figure 2:**
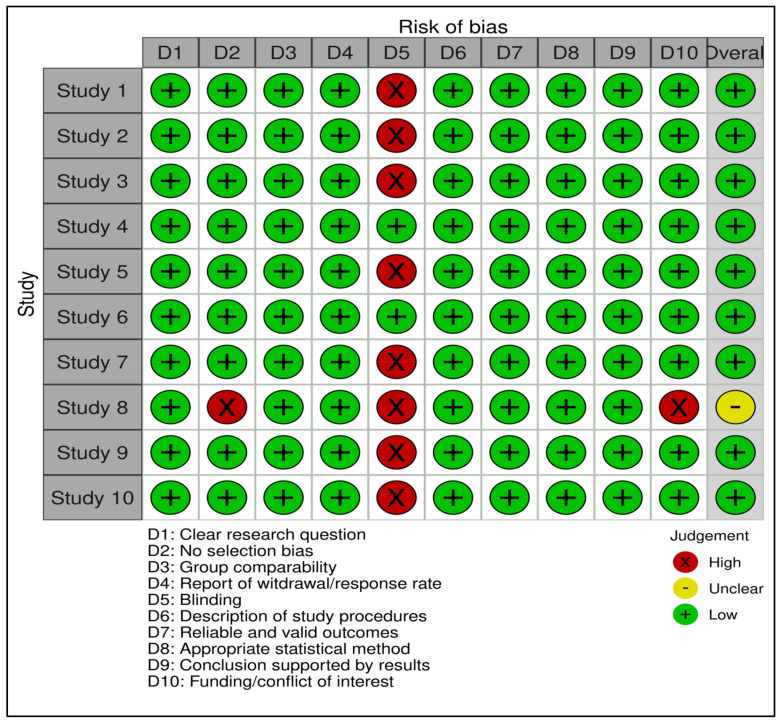
Quality appraisal for selected studies [22,23,24,25,26,27,28,29,30,31].

The risk of bias assessment (Rob) results are shown in Figure 2. Nine studies showed a low Rob, while only one study had a neutral rating due to not reporting how they controlled the bias in participants recruitment process.

### 3.3. The Association Between Aflatoxin B1 Exposure and Vitamin A Levels/Status

For the case of vitamin A, six studies (three children and three adults) assessed the association between aflatoxin exposure and vitamin A status. Among the three studies conducted on children [22,23,24], only one found a significant association between aflatoxin exposure (AF-alb > 0.80 pmol/mg) and vitamin A deficiency. However, in adult studies, all of the three studies [25,26,27], found a significant association between aflatoxin exposure (AF-alb > 2.8 pmol/mg) and vitamin A deficiency.

Moreover, all the studies that assessed the vitamin A status/levels used an observational study design, namely four of them [23,25,26,27], used the cross-sectional study design while two [22,24] used a longitudinal study design, whereas the Gong et al. [22] study followed participants in three subsequent times, and Watson et al. [24] assessed the association during harvest and post-harvest periods since both periods contribute to the AFB1 levels. Although these studies utilized an observational design, only two included sample sizes of fewer than 200 participants.

The threshold used in assessing the vitamin A status/levels differs among the studies, whereas two studies conducted on children under five years old [22,24] categorized children having a serum retinol of <0.70 μmol/L as vitamin A deficiency, and a serum retinol of >0.70 μmol/L as normal, while two [23,27] studies assessed the correlation between AFB1 levels and serum retinol levels. In addition, two adult studies [25,26] categorized serum retinol values of <0.7 μmol/L, respectively, as cut-off points for vitamin A deficiency in adults.

### 3.4. The Association Between Aflatoxin B1 Exposure and Vitamin E Levels/Status

In four studies [24,25,26,27] that assessed the association between AF-alb and vitamin E status, only one study [27] found a significant negative correlation between levels of AF-alb and vitamin E (*p* < 0.023). High serum AF-alb concentrations (median 0.985 pmol/mg) were found in subjects with low levels of vitamin E as compared with the low AF-alb concentrations (median 0.741 pmol/mg) in adults with high levels of vitamin E. In addition, all studies that assessed the vitamin E levels/status used an observational study design, with the majority of them exploring via a cross-sectional design. Notably, only one study [27] that reported a significant association between aflatoxin B1 and vitamin E had a larger sample size (>500 participants) compared to other studies, which might have contributed to the detection of the association by increasing the statistical power, reducing variability, and providing a precise estimate.

### 3.5. The Association Between Aflatoxin B1 Exposure and Vitamin C Levels/Status

One cross-sectional study [23] conducted on children 6–9 years old, found a significant (*p* < 0.01) decrease in vitamin C levels among young children with high AF-alb (>0.3 pmol/mg).

### 3.6. The Association Between Aflatoxin B1 Exposure and Zinc Levels/Status

Among the three observational studies [22,23,24] that assessed the relationship between aflatoxin B1 exposure and zinc status among children, only one study [24] found a significant association (*p* < 0.05) between children in the highest aflatoxin exposure group (>25.05 pg/mg) and those in the lowest group (<5.26 pg/mg), who were 1.98 (95%CI: 1.00, 3.92) times more likely to be zinc-deficient (serum zinc < 9.9 μmol/L) during the harvest season.

### 3.7. The Association Between Aflatoxin B1 Exposure and Low Haemoglobin Levels

Of the four studies [28,29,30,31] that assessed the association between aflatoxin exposure and low haemoglobin levels (<11 g/dL) in pregnant women, only two studies [28,31] reported an increased risk of having haemoglobin levels below the threshold (<11 g/dL) with the highest AF-alb levels (>1.13 pmol/mg). Most importantly, these studies had sample populations larger than 600, which increases the statistical power with the regards to the study design used.

## 4. Discussion

To the best of our knowledge, this is the first review to systematically review the evidence on the association between aflatoxin exposure in any micronutrient level/status in human populations. The RoB assessment revealed that only one study had a moderate risk of bias. Moreover, four studies out of six consistently found a significant positive association between aflatoxin exposure and vitamin A deficiency.

This review has highlighted that aflatoxin exposure might increase vitamin A and E deficiencies in adults and children in observational studies. Moreover, all of the studies that were conducted on adults found a positive significant association between aflatoxin and vitamin A deficiency [25,26,27]. This result can be explained by the presence of vitamin A supplementation programmes targeting children under five [32], which effectively reduces the prevalence of vitamin A deficiency in this age group. These programmes likely mitigate the impact of aflatoxin exposure on vitamin A status in children. However, such interventions are not typically implemented for adults, leaving them more vulnerable to the effects of aflatoxin on vitamin A levels/status, which could explain the observed significant association in adults but not in children.

Although all of the three adult studies from Ghana found a significant difference between aflatoxin exposure and vitamin A deficiency, the studies used a cross-sectional study design, which might have missed important factors such as seasonality [33] and could have had an impact on the vitamin A status and aflatoxin exposure. Moreover, Ghana’s tropical climate, characterized by high humidity and temperatures, creates an ideal environment for the growth of *Aspergillus flavus*, the fungus responsible for producing aflatoxins [34]. In addition, maize is a staple in Ghana, consumed more extensively than in other West African countries [35]. This higher consumption increases the level of exposure and risk of contamination compared to countries where dietary reliance on maize is less pronounced.

Although only one study assessed the association between aflatoxin exposure with vitamin C, the study found a positive significant association. There is limited evidence on the mechanism between aflatoxin exposure and micronutrient deficiencies; Benkerroum’s study [36] explained the possible mechanism by which aflatoxin might lead to these vitamin deficiencies, whereby aflatoxin can interfere with the absorption of minerals and vitamins in the gastrointestinal tract, especially vitamins A, C, and E and selenium, preventing their absorption in the intestine [36]. In addition, exposure to aflatoxins can lead to oxidative stress by producing reactive oxygen species (ROS), which can deplete antioxidants such as vitamin C. Moreover, heightened oxidative stress may surpass the body’s antioxidant defences, further reducing vitamin C levels [37].

Only one of three studies found a significant association between aflatoxin exposure and zinc deficiency. However, the potential implications of this research are significant, and more evidence is needed to fully evaluate this association. Animal studies have shown a possible mechanism through which AFB1 can damage the intestinal lining (i.e., villus atrophy), leading to impaired mineral absorption, including zinc. This is due to AFB1 disrupting tight junctions in the gut, increasing intestinal permeability and reducing the efficiency of zinc transporters (i.e., ZIP and ZnT proteins) responsible for zinc absorption [38,39,40,41].

Furthermore, this review highlighted that aflatoxin exposure might lead to anaemia or reduce haemoglobin levels in pregnant women. This finding was in line with a previous review [42] that highlighted aflatoxin as one of the factors associated with anaemia/low haemoglobin levels in several animal species. In addition, animal studies have highlighted the three mechanisms by which aflatoxin might lead to low haemoglobin levels/anaemia/iron deficiency. Firstly, aflatoxin can lead to the haemolysis of red blood cells and cause haemolytic anaemia [43]. Secondly, aflatoxin exposure can induce the release of inflammatory markers, such as interleukin 6 (IL-6), which can suppress the erythropoiesis process and lead to anaemia [44]. Thirdly, IL-6 production can also stimulate hepcidin hormone production, inhibiting iron absorption in the intestine or macrophages [45], potentially leading to iron deficiency anaemia.

Most importantly, the use of biomarkers for detecting aflatoxin exposure has been instrumental in highlighting the extent of the problem at both individual and community levels. Nearly all studies utilized the serum/plasma AF-alb adduct biomarker to evaluate aflatoxin B1 exposure, and only one study used the AFM_1_ biomarker in urine. The use of the AF-alb adduct biomarker increases the strength of the studies in the detection of aflatoxin exposure. This biomarker is recognized as the most sensitive for detecting aflatoxin because it can identify exposure up to three months prior [46]. Other biomarkers, like aflatoxin M1, detect exposure within 24–72 h after ingesting aflatoxin-contaminated foods [47,48]. While the use of biomarkers in assessing AF-alb provides strong evidence of exposure, the use of different analytical methods can introduce bias due to variations in sensitivity and specificity. Moreover, in our review, we found that all studies which used RIA as an analytical tool for AF-alb adduct biomarker found, a statistically significant association between aflatoxin exposure and vitamin A deficiency. Due to the limited number of studies that have used RIA for AF-alb adduct biomarker assessment in association with vitamin A deficiency, we reiterate the need for more studies to evaluate this evidence.

Notably, aflatoxin’s contamination of crops is associated with causing acute or chronic intoxications in humans, such as liver cancer [49] and several micronutrient deficiencies (i.e., low haemoglobin levels and deficiencies in vitamins A and E) reported in this review. Once micronutrient deficiency occurs in any population group, it can lead to different health problems such as immune suppression (i.e., deficiencies in vitamins A and E), loss of blood (i.e., low levels of haemoglobin and iron), growth impairment (i.e., deficiencies in iron and vitamin A), and poor cognitive development (i.e., deficiencies in zinc and vitamins A, B9, and B12) [1,2,50].

Although the studies we included primarily focused on the association between aflatoxin exposure and haemoglobin, zinc, and vitamins A, C, and E, our selection was determined by the breadth of existing research rather than pre-specified micronutrient categories. However, it is important to note that vitamin A, iron, and zinc are recognized as micronutrients of public health concern [2], and their deficiencies are linked to impaired immune function, growth, and cognitive outcomes [5,7,8]. This systematic review provides crucial evidence in the literature related to the association between aflatoxin exposure and select micronutrient status, with potential implications for public health. In this systematic review, we refrained from conducting a meta-analysis due to the limited number of studies available for each type of micronutrient and the substantial variability in findings across different groups. Another limitation is that the majority of our studies used a cross-sectional study design, which did not account for dietary, seasonality, and other environmental factors that could have influenced micronutrient deficiencies.

## 5. Conclusions

This review underscores the need for incorporating aflatoxin mitigation strategies within micronutrient interventions. This is particularly important given the association between aflatoxin exposure and anaemia and vitamin A deficiency in multiple studies. Furthermore, it highlights the need for further research to explore the interaction between aflatoxin exposure and micronutrient status and other potential confounding factors, such as dietary patterns and socioeconomic status in different seasons. Since most of the studies in this systematic review used a cross-sectional design, future research should focus on a longitudinal design to establish a cause–effect association between aflatoxin exposure and micronutrient status.

## Figures and Tables

**Table 1:** Description of the included studies.

No	Author	Country	Study Population	Sample Size	Study Design	AF Analytical Method	Biomarker Used to Assess AF Exposure	AF-alb Levels Associated with Micronutrient Deficiency	Micronutrients Included in the Study					Association Between AF and MNs Levels/Status
									Vit A	Vit E	Vit C	Zn	Hb	
1	Gong et al., 2004 [22]	Benin	Children aged 16–37 months	200	LD	ELISA	AF-alb (Blood)	AF-alb: >4.9 (pmol/mg)	√			√		-Vit A: NS -Zn: NS
2	Turner et al., 2003 [23]	Gambia	Children 6–9 years	472	CS	ELISA	AF-alb (Blood)	AF-alb: >0.3 (pmol/mg) for vitamin C.	√		√	√		-Vit C: S ** -Zn: NS -Vit A: NS
3	Watson et al., 2016 [24]	Guinea	Children aged 10–46 months	305	LD	ELISA	AF-alb (Blood)	(a) AF-alb: ≥2.8 (pmol/mg) for vitamin A.	√	√		√		(a) Harvest -Zn: S * -Vit A: S * -Vit E: NS
								(b) AF-alb: 0.52–1.17 (pmol/mg) for Zinc.						(b) Post-Harvest Vit A, Vit E, Zn: NS
4	Obuseh et al., 2011 [25]	Ghana	>16 years	305	CS	RIA	AF-alb (Blood)	AF-alb: ≥0.80 pmol/mg for vitamin A.	√	√				-Vit A: S *** -Vit E: NS
5	Obuseh et al., 2010 [26]	Ghana	Adults >19 years	147	CS	RIA	AF-alb (Blood)	AF-alb: ≥0.80 pmol/mg for vitamin A.	√	√				-Vit A: S * -Vit E: NS
6	Tang et al., 2009 [27]	Ghana	Adults 18–85 years old	507	CS	RIA	AF-alb (Blood)	AF-alb: ≥1.58 pmol/mg for vitamins A and E.	√	√				-Vit A: S * -Vit E: S *
7	Lei et al., 2021 [28]	China	Pregnant women	616	RCS	ELISA	AF-alb (Blood)	AF-alb: ≥6.03 pmol/mg for anaemia.					√	-Hb: S *
8	Murashiki et al., 2024 [29]	Zimbabwe	Pregnant women	129	CS	ELISA	AFM_1_ (Urine)	AFM_1_; >0.06 ng/mL					√	-Hb: NS
9	Passarelli et al., 2020 [30]	Tanzania	Pregnant women	1500	RCS	HPLC	AF-alb (Blood)	AF-alb: >1.39 pg/mg for anaemia					√	-Hb: NS
10	Shuaib et al., 2010 [31]	Ghana	Pregnant women	785	CS	HPLC	AF-alb (Blood)	AF-alb: ≥1.13 pmol/mg for anaemia.					√	-Hb: S *

√: Type of micronutrient included in the study; S: statistically significant; NS: not statistically significant; *: *p* < 0.05; **: *p* < 0.01; ***: *p* < 0.001; ELISA: Enzyme-Linked Immunosorbent Assay; AF-alb: aflatoxin albumin adducts; HPLC: High-Performance Liquid Chromatography; RIA: Radioimmunoassay; AFM_1_: aflatoxin M1 (biomarker in urine); AF—aflatoxin; CS: cross-sectional study design; RCS: repeated cross-sectional study design; LD: longitudinal study design; Vit A: vitamin A; Vit E: vitamin E; Vit C: vitamin C; Zn: Zinc; Hb: haemoglobin; MNs: micronutrients.

## Data Availability

The dataset used and/or analysed during the current study are available from the corresponding author on reasonable request. Data extracted from included studies will be made available upon request. The data are not publicly available as this is part of the ongoing study.

## Appendix A. Search Strategies Developed for Each Database

**Table A1:** Search strategy developed for PUBMED: Search conducted on 14 March 2023.

Steps	Search	Results
1	“child” [MeSH] OR children [tiab] OR “infant” [MeSH] OR infant [tiab] OR “child, preschool” [MeSH] OR toddlers [all fields] OR “adult” [MeSH] OR adults[tiab] OR “adolescent” [MeSH] OR adolescence[tiab] OR pregnant [all fields] OR “pregnant women” [MeSH] OR pregnant women [tiab]	144
2	“aflatoxins” [MeSH] OR aflatoxin [tiab] AND exposure [all fields] OR “mycotoxins” [MeSH] OR mycotoxin[tiab] OR “Aflatoxin B1” [MeSH] OR aflatoxin B1[tiab] OR “Aflatoxin M1” [MeSH] OR aflatoxin M1 [tiab] OR AFB_1_[all fields] OR AFM_1_[all fields] OR “aflatoxin B1, exposure” [MeSH] OR “AFB_1_” [MeSH]	29
3	micronutrients[MeSH] OR iron[tiab] OR ferritin OR transferrin[tiab] OR “Vitamin A” [MeSH] OR retinol[tiab] OR zinc[tiab] OR folate[tiab] OR “folic acid*” [tiab] OR anemia[tiab] OR vitamin A[tiab] OR “vitamin E” [MeSH] OR “ascorbic acid” [MeSH] or vitamin c[tiab] OR “anemia” [MeSH] OR anaemia [tiab] OR “Iron, Deficiency” [MeSH] OR “anemia, iron-deficiency” [MeSH] OR “hemoglobin” [MeSH] OR “Vitamin A Deficiency” [MeSH] OR “Zinc/deficiency” [MeSH] OR “Vitamin E Deficiency” [MeSH] OR “Deficiency, Ascorbic Acid” [MeSH]	2277
4	#1 AND #2 AND #3	144
5	#4 limit from 2003 to 2023	30
6	#5 limit to English	17

**Table A2:** Search strategies developed for Embase: Search conducted on 17 March 2023.

Steps	Search	Results
1	exp child/ or exp preschool child/ or exp school child/ or exp infant or exp under five years/ or exp toddler/ or exp adolescen*/ or exp young/ or exp teen/. or exp under five years/ or exp toddler/ or exp adolescen*/ or exp young/ or exp teen/	1,984,578
2	exp adult/ or exp pregnant/ or exp pregnant wom*n/ or exp women of reproductive age/ or exp boy/ or exp girl	8,289,589
4	1 or 2	10,274,167
5	exp aflatoxin/or exp mycotoxin/or exp aflatoxin exposure/or exp aflatoxin B1/or exp aflatoxin B1, exposure/or exp AFB_1_/ or exp AFM_1_/	49,185
8	4 AND 5	3352
9	iron/ec, [Endogenous Compound]	22,362
10	iron deficiency/ or iron sufficient/ ad, co,di,dm,ep,et,pc [Adequate, Complication, Diagnosis, Disease Management, Epidemiology, Etiology, Prevention]	2047
11	Anaemia/or non-anaemic/ no,co,di,dm,ep,et,pc [Normal, Complication, Diagnosis, Disease Management, Epidemiology, Etiology, Prevention]	17,803
12	((iron or ferritin or hemoglobin) and (serum or blood or plasma) and (level* or sufficien* or inadequa* or insufficien* or deficien*)).ti,ab,kw.	27,238
13	9 or 10 or 11or 12	60,705
14	Zinc/ec [Endogenous Compound]	8636
15	Zinc deficiency/or zinc sufficient/ ad,co,di,dm,ep,et,pc [Adequate, Complication, Diagnosis, Disease Management, Epidemiology, Etiology, Prevention]	599
16	(zinc and (serum or blood or plasma) and (level* or sufficien* or inadequa* or insufficien* or deficien*)).ti,ab,kw.	4993
17	14 or 15 or 16	12,792
18	Vitamin A/ ec, [Endogenous Compound]	4184
19	Vitamin A deficiency/or vitamin A sufficient/ ad,co,di,dm,ep,et,pc [Adequate, Complication, Diagnosis, Disease Management, Epidemiology, Etiology, Prevention]	519
20	((retinol or “vitamin A”) and (serum or blood or plasma) and (level* or sufficien* or inadequa* or insufficien* or deficien*)).ti,ab,kw.	2501
21	18 or 19 or 20	6392
22	Vitamin C/ec, [Endogenous Compound]	6326
23	Vitamin C Deficiency/or vitamin C sufficient/ ad,co,di,dm,ep,et,pc [Adequate, Complication, Diagnosis, Disease Management, Epidemiology, Etiology, Prevention]	217
24	((vitamin C or ascorbic acid or ascorbate) and (serum or blood or plasma) and (level*or sufficien* or inadequa* or insufficien* or deficien*)). ti,ab,kw	1714
25	22 or 23 or 24	7913
26	Vitamin E/ ec, [Endogenous Compound]	4874
27	Vitamin E Deficiency/or vitamin E sufficient/ ad,co,di,dm,ep,et,pc [Adequate, Complication, Diagnosis, Disease Management, Epidemiology, Etiology, Prevention]	229
28	((vitamin E or tocopherol) and (serum or blood or plasma) and (level* or sufficien* or inadequa* or insufficien* or deficien*)). ti,ab,kw	1351
29	26 or 27 or 28	6101
30	13 or 17 or 21 or 25 or 29	82,539
31	8 AND 30, limit to English and year from 2003–2023	100

**Table A3:** Search strategy developed for Scopus: Search conducted on 21 March 2023.

Steps	Search	Results
1	TITLE-ABS-KEY (child OR infan OR preschool OR young child OR school child OR teen OR under five year OR toddler OR adolescent OR adult OR pregnant OR pregnant wom?n OR women of reproductive age)	600
2	TITLE-ABS-KEY (aflatoxin* OR mycotoxin* OR aflatoxin exposure OR aflatoxin B1 OR aflatoxin B1, exposure OR AFB_1_ OR aflatoxin M1 OR aflatoxin M1, exposure OR AFM_1_)	7952
3	#1 AND #2	55
4	TITLE-ABS-KEY ((iron OR ferritin OR h*moglobin OR zinc OR vitamin A OR retinol OR Vitamin C OR ascorbic acid OR vitamin E OR tocopherol*) AND TITLE-ABS-KEY (blood OR serum OR plasma) AND TITLE-ABS-KEY (sufficien* OR inadequa* OR insufficien* OR deficien*))	1803
5	# 3 AND #4, limit to English and limit to 2003–2023	177

**Table A4:** Search strategy developed for Web of Science: Search conducted on 27 March 2023.

Steps	Search	Results
1	TS = (“child*” [tiab] OR “infan*” [tiab] OR “pre*school” [tiab] OR “young child*” [tiab] OR “school child*” [tiab] OR “teen” [tiab] OR “under five years” [tiab] OR “toddler” [tiab] OR “adolescent*” [tiab] OR “adult*” [tiab] OR “pregnant” [tiab] OR “pregnant wom*n” [tiab] OR “women of reproductive age” [tiab]	8134
2	TS = (“Aflatoxin*” [mh] [tiab] OR “mycotoxin*” [tiab] OR “aflatoxin exposure” [tiab] OR “Aflatoxin B1 Exposure” [tiab] OR “AFB_1_” [tiab] OR “Aflatoxin M_1_” OR “AFM_1_”)	24,925
3	TS = (“Low aflatoxin B1 exposure” [tiab] OR “without aflatoxin B1 exposure” [tiab] OR “no exposure” [tiab] OR “other intervention*” [tiab])	19,062
4	TS = (“micronutrient*” [mh] OR “micronutrient deficien*” [tiab] OR “vitamin*” [tiab] OR “mineral*” [tiab] OR “vitamin A” [tiab] OR “retinol” [tiab] OR “beta carotene” [tiab] OR “retinoids” [tiab] OR “iron*” [tiab] OR “h*emoglobin” [tiab] OR “iron deficien*” [tiab] OR “iron deficien*” [tiab] “an*emia” [tiab] OR “Hb” [tiab] OR “vitamin C” [tiab] OR “ascorbic acid” [tiab] OR “ascorbate” [tiab] OR “zinc” [tiab] OR “selenium” [tiab] OR “vitamin E” [tiab] OR “tocopherol*” [tiab] OR “vitamin D” [tiab] OR “calciferol” [tiab])	368
5	#1 AND #2 AND #3 AND #4, limit to English, limit to 2003 to 2023	37
